# Interaction between growing oocytes and granulosa cells in vitro

**DOI:** 10.1002/rmb2.12292

**Published:** 2019-08-22

**Authors:** Md Hasanur Alam, Takashi Miyano

**Affiliations:** ^1^ Department of Animal Science, Faculty of Animal Husbandry Bangladesh Agricultural University Mymensingh Bangladesh; ^2^ Graduate School of Agricultural Science Kobe University Kobe Japan

**Keywords:** BMP15, bovine oocyte, follicular antrum, GDF9, granulosa cell, transzonal projection

## Abstract

**Background:**

Oocyte growth is accompanied by follicular development in mammalian ovaries. Since the discovery of two oocyte‐derived factors, growth differentiation factor 9 (GDF9), and bone morphogenetic protein 15 (BMP15), knowledge of the bidirectional communication between oocytes and granulosa cells for ovarian function and fertility has been accumulated. In addition, the growth culture system of oocytes has been improved, further promoting the studies on the communication between oocytes and granulosa cells in vitro.

**Methods:**

We provide an overview of the role of granulosa cells in oocyte growth and the role of oocytes in follicular development along with our recent findings in culture experiments of bovine growing oocytes.

**Main findings:**

Granulosa cells supply nutrients and metabolites through gap junctions to oocytes and secrete paracrine signals to regulate oocytes. Oocytes regulate granulosa cell proliferation and differentiation and induce antrum formation *via* GDF9 and BMP15.

**Conclusion:**

Oocytes actively participate in various aspects of follicular development, including antrum formation *via* the oocyte‐derived factors GDF9 and BMP15, whose synthesis is probably regulated by granulosa cells. In vitro studies will reveal the precise communication loop between oocytes and granulosa cells that facilitates the coordinated development of oocytes and granulosa cells in the follicles.

## 1 INTRODUCTION

**Figure 1:**
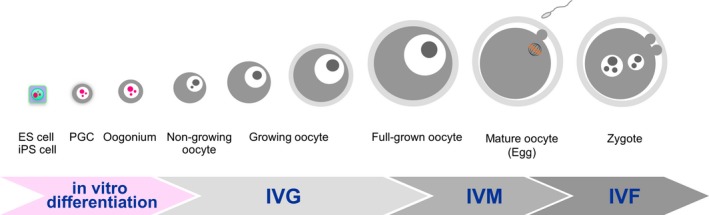
Schematic flow of the in vitro differentiation of ES cells and iPS cells, and in vitro growth (IVG), in vitro maturation (IVM), and in vitro fertilization (IVF) of mammalian oocytes. ES cell: embryonic stem cell; iPS cell: induced pluripotent stem cell; and PGC: primordial germ cell

Mammalian oogenesis starts at the embryonic period, and primordial germ cells (PGCs) are the primary cells in the process (Figure 1). After migration to the embryonic gonads, PGCs become oogonia and proliferate by mitosis. The oogonia subsequently enter meiosis I, at which point they are called oocytes, and become arrested at the diplotene stage of meiosis I.^1^ Individual oocytes become enclosed by a single layer of flattened pre‐granulosa cells in primordial follicles.^2^ When the oocytes start to grow, the surrounding pre‐granulosa cells become cubic granulosa cells in the primary follicles.^3^ The granulosa cells proliferate and form a multilayered structure, which is then further surrounded by layers of theca cells. Follicles at this stage are called secondary follicles. Later, a fluid‐filled cavity is formed inside the follicles and they become antral follicles. At this stage, the granulosa cells differentiate to cumulus granulosa cells which enclose the oocytes, and mural granulosa cells which form the inner layer of the follicle wall.^4^ In the meantime, the oocytes increase in size and prepare themselves for future maturation and fertilization with spermatozoa. After female animals reach puberty, the periodic gonadotropic surge induces fully grown oocytes in the antral follicles to resume meiosis I, to mature to metaphase II (MII), and finally to be ovulated.

The development of follicles is regulated by the hormones synthesized at the different levels in the hypothalamic‐pituitary‐ovarian axis: The hypothalamus secretes gonadotropin‐releasing hormone, the anterior pituitary secretes follicle‐stimulating hormone (FSH) and luteinizing hormone (LH), and the ovary produces steroidal hormones.^5^ In the follicles, FSH stimulates granulosa cell proliferation and aromatization of androgens to estrogens. Estrogens also stimulate granulosa cell proliferation.^6^ Previous studies have demonstrated that FSH receptors are expressed in the follicles from the primary to later stages 7 and that treatment with FSH promotes development of preantral follicles.^8^ Other studies have analyzed the growth‐promoting 6 and anti‐apoptotic actions 9 of FSH in the antral follicles. It has long been known that FSH is the predominant regulator of follicular development.

In addition to the endocrinological studies, ultrastructural studies have revealed the presence of direct connections between oocytes and granulosa cells. Anderson and Albertini showed the presence of heterologous gap junctions between oocytes and granulosa cells.^10^ Further, demonstration of lucifer yellow dye transfer from oocytes to granulosa cells clearly showed that there is direct communication through the gap junctions.^11^ Through the gap junctions, granulosa cells efficiently provide small molecules, such as nutrients, metabolic precursors, and molecular signals that regulate the oocytes.^12^ Therefore, prior to the discovery of the oocyte‐derived growth factors, it was thought that oocyte growth was controlled unidirectionally, with the surrounding granulosa cells supporting oocytes nutritionally, and FSH regulating the proliferation of granulosa cells.

In 1977, Eppig performed the first systematic study of in vitro growth (IVG) culture of oocytes,^13^ and it has since undergone continuous development as a new technology for utilizing incompetent oocytes in the ovary as a source of mature oocytes (Figure 1). In mice, application of IVG culture was extended from growing oocytes in secondary follicles 13 to non‐growing oocytes in primordial follicles in 1996,^14^ and to the production of baby mice from oogonia in fetal gonads in 2016.^15^ Further, Saitou and his colleagues produced PGC‐like cells from embryonic stem cells (ES cells) and induced pluripotent stem cells (iPS cells) derived from embryonic fibroblasts and adult tail tip fibroblasts,^16^, ^17^ and Hayashi and his colleagues generated fully potent mature oocytes completely in culture from ES cells and iPS cells.^18^ Our group has focused on IVG culture of domestic species, which would provide a new source of mature eggs for livestock production by using existing assisted reproductive technologies, such as in vitro maturation (IVM) and in vitro fertilization (IVF) of oocytes. Although progress on these technologies is far behind that of their counterparts in mice, the culture systems have been improved, and now, bovine growing oocytes collected from early antral follicles are able to grow to their final size and acquire the full developmental capacity efficiently.^19^ In both mice and domestic species, oocyte‐granulosa cell complexes are cultured, and most researchers have tried to maintain the granulosa cell viability and oocyte‐granulosa cell attachment during the long‐term culture period, because direct association of surrounding granulosa cells with oocytes through gap junctions is crucial for the oocyte viability and growth in vitro.

In addition to the endocrinological control of follicular development and the support provided to oocytes by granulosa cells, a new group of players joined the field of follicular development about two decades ago. These are the oocyte‐derived growth factors: growth differentiation factor 9 (GDF9) and bone morphogenetic protein 15 (BMP15).^20^, ^21^ Oocytes carry on their conversation with these factors inside the follicles, and follicular development is under the bidirectional communication between oocytes and granulosa cells.^22^ Now, a bovine IVG system for growing oocytes is nearly established.^23^, ^24^ Just as IVF in different species has provided a new understanding of mammalian fertilization and IVM has elucidated many aspects of the molecular control mechanisms of oocyte maturation, IVG of oocytes is expected to provide a new understanding of the mechanisms regulating the complex process of follicular development and oocyte growth in the mammalian ovary.

In this review, the bidirectional communication between oocytes and granulosa cells is outlined. We briefly review the role of granulosa cells in oocyte growth and the role of oocytes in follicular development, and reveal a new role of oocytes in antrum formation *via* oocyte‐derived growth factors based on our recent findings in bovine IVG experiments. For an explanation of the IVG culture systems used in our study, we recommend the reviews of Hirao.^23^, ^24^, ^25^

## 2 GRANULOSA CELLS FOR OOCYTE GROWTH

### 2.1 Structural connection between oocytes and granulosa cells

**Figure 2:**
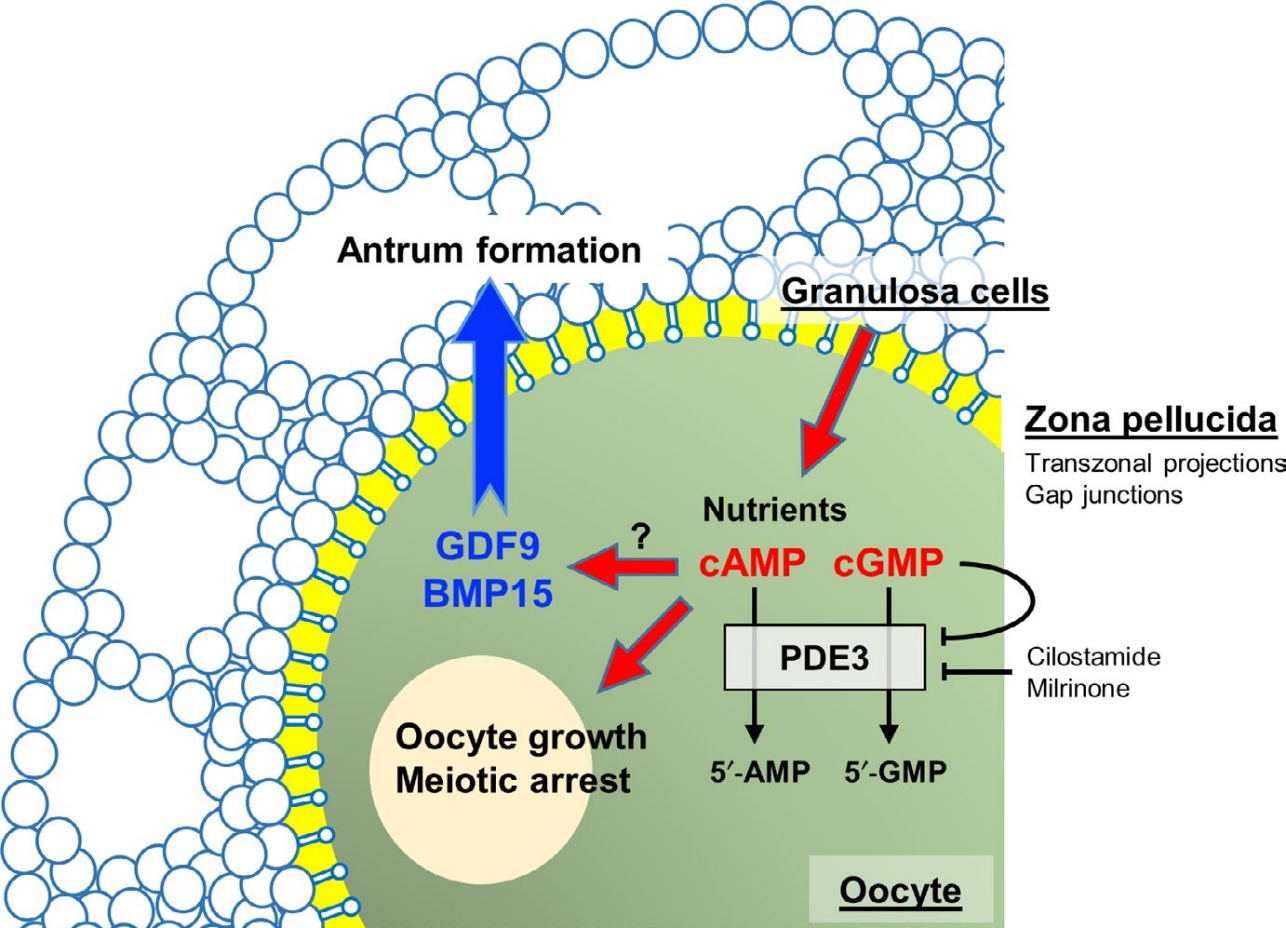
Schematic model of communication between oocytes and granulosa cells. Nutrients, cAMP, and cGMP are transported from granulosa cells through their transzonal projections and gap junctions to oocytes to support the growth and meiotic arrest of oocytes. The oocyte‐derived growth factors GDF9 and BMP15 promote granulosa cell proliferation and differentiation, and antrum formation. Inhibition of oocyte PDE3 increases the expressions of GDF9 and BMP15, which in turn promote antrum formation. PDE3: phosphodiesterase 3; cAMP: cyclic adenosine 3′, 5′‐monophosphate; cGMP: cyclic guanosine 3′, 5′‐monophosphate; 5′‐AMP: 5′‐adenosine monophosphate; 5′‐GMP: 5′‐guanosine monophosphate; and PDE3 inhibitors: cilostamide and milrinone

In primordial follicles, non‐growing oocytes are directly adjacent to surrounding pre‐granulosa cells. Shortly after oocytes enter the growth phase, an extracellular coat called the zona pellucida is assembled around the oocytes. Even after zona pellucida formation, however, the granulosa cells maintain contact with the oocytes *via* cytoplasmic processes known as transzonal projections (TZPs), which penetrate the zona pellucida (Figure 2). TZPs originate from granulosa cells, and some of them terminate at the oolemma to provide a means of direct connection between oocytes and granulosa cells.^10^, ^26^, ^27^ Most TZPs are composed of a strong backbone made of actin filaments,^28^ whereas a much smaller number of TZPs contain tubulin.^29^ Multiple TZPs typically project from each granulosa cell adjacent to the zona pellucida, while long actin‐rich filaments also project from some granulosa cells located in layers more distal to the oocyte.^27^, ^30^, ^31^ A dynamic change in the number and shape of TZPs occurs during follicular development. In growing oocytes, numerous TZPs develop and contribute to the growth. However, during the maturation of fully grown oocytes following the gonadotropic surge, active retraction of TZPs has been noted.^32^ In IVG of growing oocytes from domestic species, the number of TZPs significantly decreased during the culture, although the decrease was prevented by estradiol 17β for bovine oocytes,^33^ and by FSH for porcine oocytes.^34^

At the tip of TZPs, granulosa cells form heterologous gap junctions with oocytes. Gap junctions are intercellular channels that permit the direct transfer of ions and small molecules (<1 kDa) between adjacent cells.^35^ Gap junction channels are composed of connexins (Cx), a family of more than 20 members.^36^ Six connexins oligomerize to form a connexon (gap junction hemichannel), and two connexons in adjacent cells (between oocyte and granulosa cells, and between granulosa cell and granulosa cell in the follicle) dock to make a channel between the cells.^37^ Ovarian follicles of rodents express Cx32, Cx37, Cx43, and Cx45.^38^ Cx43 expression is restricted to the granulosa cells, whereas Cx37 is expressed exclusively in the oocytes.^39^ Bovine follicles express Cx26, Cx32, Cx37, and Cx43 40, 41; Cx43 is localized in granulosa cells 41, 42; and Cx26 is detected in oocytes.^40^ In the bovine follicles, Cx37 is expressed in both the oocytes and granulosa cells.^42^

Gap junctional channels transfer lucifer yellow dye, radiolabeled uridine metabolites, and electrical current in hamster oocyte‐granulosa cell complexes.^11^ Other studies suggested that granulosa cells provide nutrients, metabolic precursors, and signaling molecules through gap junctional channels to the oocytes.^12^, ^26^ Moreover, granulosa cells are coupled together *via* homologous gap junctions, so that the whole follicle including the oocyte, but not the theca cells, which are separated by the basement membrane, makes a functional syncytium.^43^ Small molecules such as amino acids, nucleotides, metabolites, and cyclic adenosine 3′,5′‐monophosphate (cAMP) are also exchanged through gap junctions among granulosa cells. Mice lacking Cx37 never develop mature Graafian follicles.^44^, ^45^ Cx37 deletion causes oocytes to arrest their growth at 74% in diameter of normal size and fail to achieve full meiotic competence.^45^

### 2.2 Amino acids and energy substrates

Through heterologous gap junctions, granulosa cells transport nutrients such as amino acids 46, 47 and substrates for energy production 48 to the oocytes. Six amino acid transport systems have been identified in mouse growing oocytes.^49^ Although the presence of granulosa cells surrounding growing oocytes does not confer amino acid transport by additional transport systems not present in the oocytes, the granulosa cells enhance the uptake of glycine, alanine, lysine, and taurine by oocytes, perhaps *via* gap junctions.^49^

Glucose uptake by oocytes occurs *via* the facilitative glucose transporter (GLUT) proteins in mice,^50^ cows,^51^ sheep,^52^ humans,^53^ and rhesus monkeys.^54^ But mammalian oocytes have low capacity to utilize glucose as a substrate,^50^, ^55^, ^56^, ^57^, ^58^, ^59^ possibly due to having a limited amount of the glycolytic enzyme phosphofructokinase.^60^ Thus, oocytes in most mammalian species appear to rely on granulosa cells that contain an additional GLUT with high affinity to glucose and high phosphofructokinase activity to convert glucose into readily utilized substrates (ie, pyruvate, lactate, NADPH *etc*).^55^, ^58^ These substrates are used by oocytes for the energy metabolism necessary for oocyte growth.^59^ Oocytes denuded of their granulosa cells are able to utilize pyruvate and other intermediaries of the tricarboxylic acid pathway for energy production, but not glucose.^60^

Granulosa cell–free culture systems have been reported in which naked mouse oocytes grew to 35 µm in diameter and formed the zona pellucida, and some of the oocytes reached around 70 µm in the existence of cocultured thecal stem cells.^61^ However, in general, the rate of oocyte growth in vitro is directly correlated with the number of granulosa cells coupled to a given oocyte.^26^ In domestic species, growing oocytes normally become degenerated when they detach from granulosa cells in IVG culture.^25^ Direct association with granulosa cells supports oocyte viability and growth through the gap junctions, which serve as efficient passages for amino acids and energy substrates.

### 2.3 cAMP and cGMP

During the growth phase, oocytes are arrested at the prophase of meiosis I. After reaching their full size, oocytes in the large antral follicles resume meiosis in response to the FSH + LH surge. Small oocytes in the primordial, primary, and secondary follicles (even in the early antral follicles in large mammals) have no ability to resume meiosis.^62^ During the final growth phase, oocytes acquire meiotic competence in a stepwise manner; first, they acquire the competence to resume meiosis and then become competent to progress to MII.^63^ However, after oocytes become competent, they do not resume meiosis in the follicle until being stimulated by the gonadotropic surge. Meiotic resumption of oocytes is prevented by the inhibitory influence of the follicular environment, mainly by inhibitory substances produced by granulosa cells. Thus, after such competent oocytes are released from the follicle environment, they resume meiosis spontaneously without gonadotrophic hormones,^64^ especially after denudation of the surrounding granulosa cells.^65^

Spontaneous meiotic resumption of isolated oocytes is proceeded by a drop in intracellular levels of cAMP.^66^ Several studies have suggested that cAMP derived from cumulus granulosa cells maintains the meiotic arrest of oocytes 67, 68 (Figure 2). Another cyclic nucleotide, cyclic guanosine 3′,5′‐monophosphate (cGMP), also plays a role in the maintenance of oocyte meiotic arrest. cGMP passes through gap junctions into the oocytes, where it inhibits phosphodiesterase 3 (PDE3), a hydrolytic enzyme of cAMP. It has been well established in mice that the inhibition of PDE3 maintains a high concentration of cAMP in the oocytes in order to block the meiotic resumption.^69^ These cyclic nucleotides act as negative regulators of the meiotic resumption of oocytes.

cAMP is synthesized by adenylyl cyclase, and cGMP is synthesized by guanylyl cyclase. cAMP is degraded to 5′‐AMP, and cGMP is degraded to 5′‐GMP by a group of enzymes known as phosphodiesterases (PDEs). Earlier experiments to assess the effects of inhibiting PDE activity on the meiotic resumption of cumulus‐enclosed and denuded, fully grown oocytes were performed with 3‐isobutyl‐1‐methylxanthine (IBMX), a non‐specific inhibitor of PDEs. This inhibitor prevented the meiotic resumption of oocytes in different species, including rodents 70 and cows.^71^ Specific inhibition of PDE3 family members, but not of PDE4 isoforms, prevented the spontaneous maturation of rat,^72^ mouse,^73^ and porcine 74 oocytes in vitro. In bovine oocytes, PDE3 inhibition delayed meiotic maturation and increased cAMP levels.^75^

The predominant guanylyl cyclase present in granulosa cells is natriuretic peptide receptor 2 (NPR2),^76^ a receptor whose activity is stimulated by a ligand called natriuretic peptide type C (NPPC; also known as CNP). Treatment of isolated cumulus‐oocyte complexes (COCs) with NPPC promotes elevation of cGMP levels.^77^ Moreover, mutations in either the *Npr2* or *Nppc* gene in mice result in a failure to maintain meiotic arrest, leading to the precocious meiotic resumption of oocytes.^77^ Thus, the NPPC/ NPR2 system for generating cGMP in cumulus cells is crucial for the maintenance of meiotic arrest of oocytes.^78^

Downs and Eppig have reported that cAMP and a low‐molecular‐weight factor in pig follicular fluid (PFF) act synergistically to maintain meiotic arrest of mouse oocytes.^79^ Subsequent studies identified hypoxanthine as the principal molecule responsible for the inhibitory action of PFF.^80^ Hypoxanthine has cAMP‐phosphodiesterase‐inhibiting activity and maintains the meiotic arrest of fully grown mouse 81 and porcine oocytes.^82^ Moreover, it works beneficially in IVG cultures for porcine 83 and bovine growing oocytes.^84^

### 2.4 Paracrine factors

It is well known that granulosa cells synthesize estrogens which stimulate proliferation of granulosa cells.^85^ Despite the clear evidence that contact with granulosa cells is required for oocyte growth, few granulosa cell–derived paracrine growth factors have been shown to promote this process directly. Granulosa cells synthesize activin and inhibin, both of which are members of the transforming growth factor‐β (TGF‐β) superfamily and regulate FSH secretion from the pituitary gland,^86^ although the direct actions of these hormones on oocyte growth seem to be limited.^87^

The most studied ligand‐receptor system to be characterized for its role in mediating granulosa‐oocyte interactions is KIT, a receptor tyrosine kinase, and its ligand, KIT ligand (KL; this ligand is also known as stem cell factor). KIT is expressed by oocytes at all stages of follicular development (as shown in mice 88 and humans 89), and KL is expressed in granulosa cells in various mammalian species (rats,^90^ mice,^91^ and humans 92). In mice, KL has been shown to stimulate oocyte growth,^61^, ^93^ and in vitro studies support the possible requirement of KIT/KL for the initiation of follicular development.^94^ Although some studies have suggested that oocytes grew and follicles developed without KIT signaling, KIT is essential for the survival of oocytes 95, 96 and may control the reawakening of dormant oocytes in primordial follicles.^97^

## 3 OOCYTES FOR FOLLICULAR DEVELOPMENT

### 3.1 Oocyte‐derived factors: GDF9 and BMP15

Oocyte‐derived factors that directly affect granulosa cell function were predicted by several oocyte‐ectomy (removal of oocytes) experiments. The findings that the experimental removal of oocytes from rabbit ovarian follicles resulted in luteinization of granulosa cells,^98^ and that oocytes prevented the luteinization of cultured rat granulosa cells 99 suggested that some factors coming from oocytes might prevent spontaneous luteinization of granulosa cells and control their endocrine function. These experiments were based on the physiological phenomenon that ovulation triggered the luteinization of granulosa cells. In 1990, Eppig's group provided much clear evidence of the direct effect of oocytes on granulosa cell function in experiments using cultured mouse oocyte‐cumulus granulosa cell complexes.^100^, ^101^ They removed oocytes from oocyte‐cumulus granulosa cell complexes by a micromanipulation and found that FSH‐induced mucification and expansion of the complexes required the presence of oocytes. They postulated that mouse oocytes secreted a factor called “cumulus expansion‐enabling factor.” It was also found that bovine 102 and porcine 103 oocyte‐ectomized cumulus granulosa cell complexes were able to expand by the FSH stimulation. We cultured porcine growing oocyte‐cumulus‐granulosa cell complexes and found that oocytes induced the formation of follicular antrum‐like structures in vitro.^104^ Active role of oocytes in controlling glycolysis and activity of the tricarboxylic acid (TCA) cycle in granulosa cells have also been established using mouse oocyte‐ectomized complexes.^105^ It is clear that oocytes secrete some specific factor(s) that play key roles in controlling the function of granulosa cells.

In the late 1990s, several papers reported solid evidence of the presence of two oocyte‐derived growth factors: GDF9 20 and BMP15.^21^ These studies were the first to show that mammalian oocytes produce specific growth factors that regulate follicular development. GDF9‐deficient mice develop primordial follicles, but follicular development is arrested at the stage with one or two layers of granulosa cells, which leads to complete infertility.^20^ BMP15‐null female mice are subfertile and usually have minimal ovarian histopathological defects, but demonstrate decreased rates of ovulation and fertilization.^21^ GDF9 is essential for normal folliculogenesis in sheep.^106^ Immunization against GDF9 and BMP15 reduced antral follicles in cattle.^107^

Now, the predicted “cumulus expansion‐enabling factor” in mice has been identified as GDF9 itself,^108^ and it has been revealed that mouse oocytes control energy production by granulosa cells *via* oocyte‐derived BMP15.^109^ Both GDF9 and BMP15 are TGF‐β superfamily members, and the specific expression of the proteins or transcripts in oocytes has been reported in various mammalian species including mice,^20^, ^110^, ^111^ rats,^112^, ^113^, ^114^ cows,^115^ sheep,^115^, ^116^ and humans.^117^ The essential roles of GDF9 and BMP15 in regulating the differentiation and function of granulosa cells in the mouse have been studied using cultured granulosa cells and IVG culture of growing oocytes.^118^, ^119^

### 3.2 Proliferation and morphodynamics of granulosa cells

Each primordial follicle consists of an oocyte and a surrounding single layer of flattened granulosa cells. Once the oocytes start to grow, granulosa cells change their morphology from a flattened to cuboidal shape and proliferate throughout the subsequent follicular development.^3^ It is well known that proliferation of granulosa cells is stimulated by FSH and estradiol 17β in vivo and in vitro.^120^ It was also demonstrated in culture experiments that mouse oocytes stimulate the proliferation of granulosa cells from preantral follicles and the proliferation of more differentiated cumulus and mural granulosa cells from antral follicles.^121^ As expected from the phenotype in GDF9‐knockout mice, whose folliculogenesis was retarded,^20^ GDF9 is one of the factors for proliferation of granulosa cells.^112^ Recent studies using recombinant GDF9 and BMP15 have also shown that these growth factors stimulate proliferation of cultured granulosa cells in rats,^122^, ^123^ sheep,^124^ and cattle.^124^

Morphodynamics study of human antral follicles revealed rhomboid‐shape cumulus cells extending microvilli.^32^ Granulosa cells change their morphology during in vitro culture. They showed a fibroblast‐like appearance in the culture dish. This transformation of granulosa cells is modified by FSH, cAMP analogs, and growth factors.^125^ Rat granulosa cells show an epithelial shape in the presence of FSH or an adenylyl cyclase activator, cholera toxin,^126^ and they exhibit a round shape with cellular projections in the presence of 8‐bromo‐cAMP.^125^

**Figure 3:**
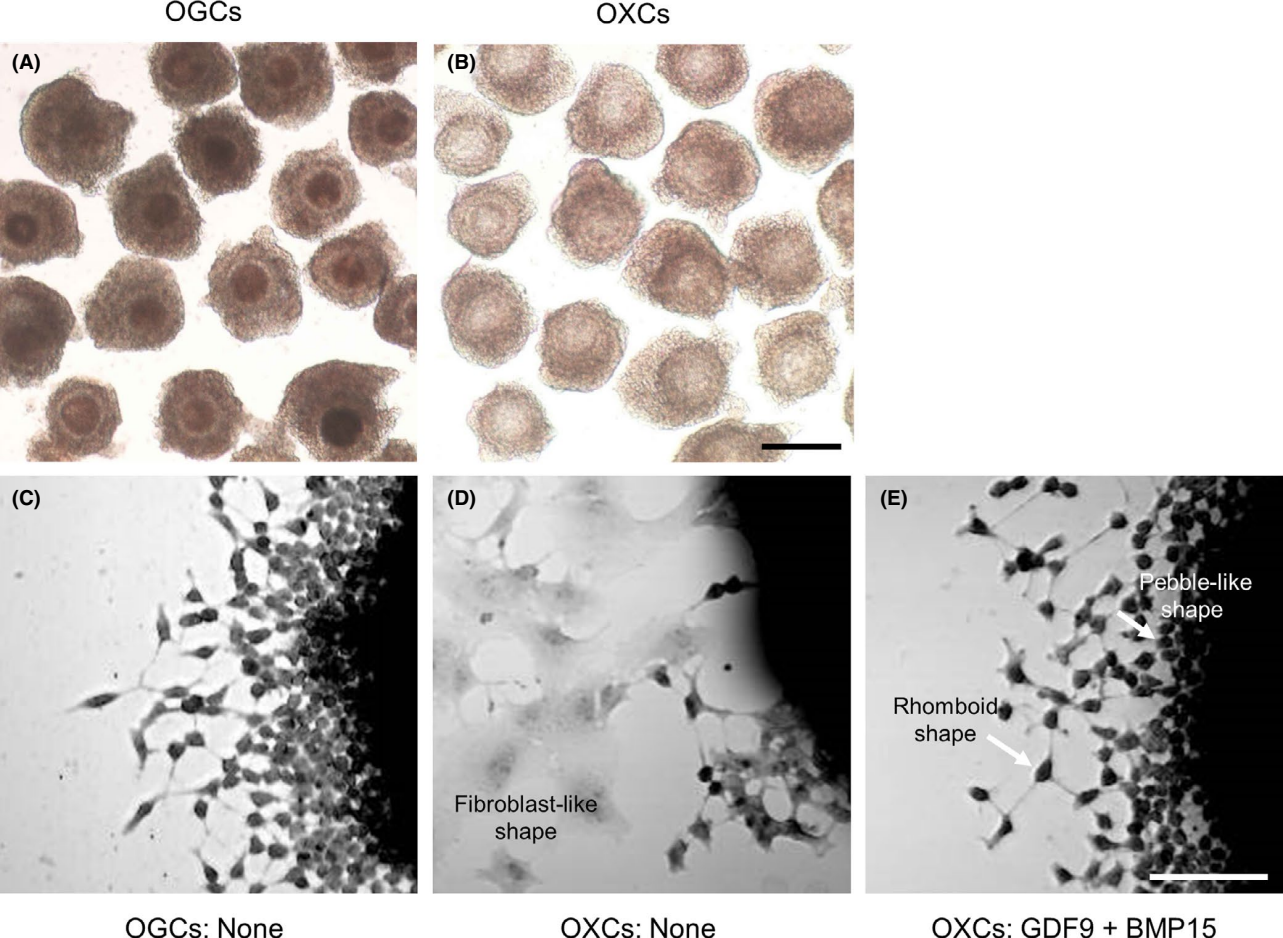
Preparation and culture of bovine oocyte‐granulosa cell complexes (OGCs). OGCs containing growing oocytes (A) were collected from small antral follicles (1.2–1.8 mm in diameter). From OGCs, oocyte‐ectomized complexes (OXCs: B) were prepared by removing the oocyte cytoplasm with the germinal vesicle. After 5 days of culture, the complexes were fixed and stained with hematoxylin and eosin Y (C–E). In OXCs (D), outgrowing granulosa cells differentiated into fibroblast‐like cells. GDF9 and BMP15 reduced fibroblast‐like cells in OXCs, while rhomboid‐shaped and pebble‐like cells were observed (E), which were similar to those in control OGCs cultured without these factors (C). The scale bar in A and B represents 200 μm; that in C–E represents 50 μm

Recently, we examined the effects of GDF9 and BMP15 on the morphology of cultured bovine growing oocyte‐granulosa cell complexes (OGCs).^127^ From OGCs, we prepared oocyte‐ectomized complexes (OXCs) and granulosa cell complexes without oocytes (GCs) to elucidate the effect of these growth factors (Figure 3). In OXCs and GCs cultured without GDF9 and BMP15 or with BMP15 alone, outgrowing granulosa cells differentiated into fibroblast‐like cells (Figure 3D). The combination of GDF9 and BMP15 suppressed the appearance of fibroblast‐like cells in OXCs and GCs, causing the granulosa cells to appear rhomboid and pebble‐like in shape (Figure 3E), much like OGCs cultured without GDF9 and BMP15 (Figure 3C). Moreover, the rhomboid cells were connected to each other by long, thin cytoplasmic projections resembling filopodia. These results suggested that oocytes maintain the granulosa cell morphology *via* GDF9 and BMP15, and that GDF9 and BMP15 might promote the generation of filopodia in outgrowing granulosa cells which change the morphology to a rhomboid shape.^127^ Another recent study indicated that GDF9 derived from mouse growing oocytes induced granulosa cells to generate specialized filopodia, which penetrated the zona pellucida (TZPs) and provided a foundation for oocyte‐granulosa cell communication.^30^ Baena and Terasaki proposed an interesting model in which “default” granulosa cells become cumulus cells if they contact the oocytes through their cytoplasmic projections (TZPs) and receive the GDF9 signal from the oocytes, based on their observation that all granulosa cells in the follicle extend many cytoplasmic projections orienting in many directions.^27^ Kossowska‐Tomaszczuk et al reported that human‐derived granulosa cells were differentiated into other cell lineages, such as osteoblasts, chondrocytes, and neurons,^128^ and Oki et al reported that porcine mural granulosa cells underwent differentiation into osteoblasts.^129^ Granulosa cells are probably differentiable cells, and oocyte‐derived growth factors may be required for them to maintain their original characteristics in the follicle.

### 3.3 Antrum formation

In the late stage of follicular development, small fluid‐filled follicular antra are formed in the granulosa cell layers and the antra fuse together into a single large antrum. The follicular antrum is a mammalian‐specific structure, which is not formed in oviparous animals, and perhaps has an important role in follicular selection in the ovary and mammalian viviparity. As IVG culture methods developed, many researchers found that the preantral follicles and growing oocyte‐granulosa cell complexes from different mammalian species (mice,^130^ pigs,^131^ and cows 84) formed antrum‐like structures in vitro. Gore‐Langton and Daniel cultured rat preantral follicles and found that FSH stimulated antrum‐like reorganization of the granulosa cells.^132^ In their report, they observed that preantral follicles, which inadvertently lost oocytes did not form antrum‐like structures, and suggested that oocytes may participate in antrum formation.

**Figure 4:**
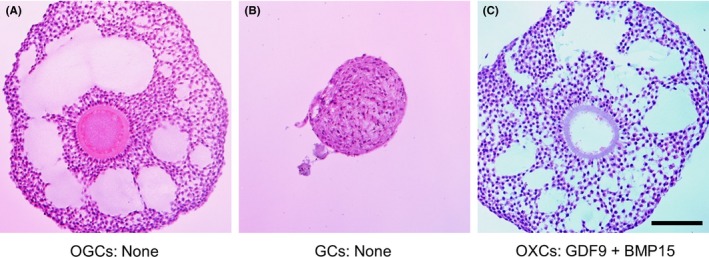
Representative images of histological sections from cultured bovine growing oocyte‐granulosa cell complexes (OGCs). OGCs containing growing oocytes were collected form bovine small antral follicles as described in the footnote of Figure 3. From OGCs, oocyte‐ectomized complexes (OXCs) and granulosa cell complexes (GCs) without oocytes were prepared. After culture, the complexes were fixed, sectioned, and stained with hematoxylin and eosin Y. OGCs developed antrum‐like structures in vitro (A), whereas GCs (B) and OXCs (not shown) did not. However, OXCs (C) developed antrum‐like structures when they were cultured with GDF9 and BMP15. The scale bar represents 100 μm

Clear evidence of a role of oocytes in antrum formation was reported by Shen et al based on an IVG culture of oocyte‐cumulus‐granulosa cell complexes collected from porcine early antral follicles.^104^ When the oocytes surrounded by cumulus cells in the complexes were replaced by denuded oocytes or Sephadex G‐25 beads, the complexes with denuded oocytes formed antrum‐like structures, whereas the complexes with beads did not. This result suggested that oocytes secreted some factor(s) inducing antrum formation. We recently used bovine oocyte‐granulosa cell complexes to examine the effects of GDF9 and BMP15 on the formation of antrum‐like structures.^127^ OXCs and GCs without oocytes did not develop any antrum‐like structure. However, GDF9 or BMP15 induced antrum‐like structures in OXCs and GCs; moreover, the combination of GDF9 and BMP15 was more potent for the formation of antrum‐like structures in these complexes (Figure 4). These results suggest that oocytes induce granulosa cells to form the antrum *via* GDF9 and BMP15.

**Figure 5:**
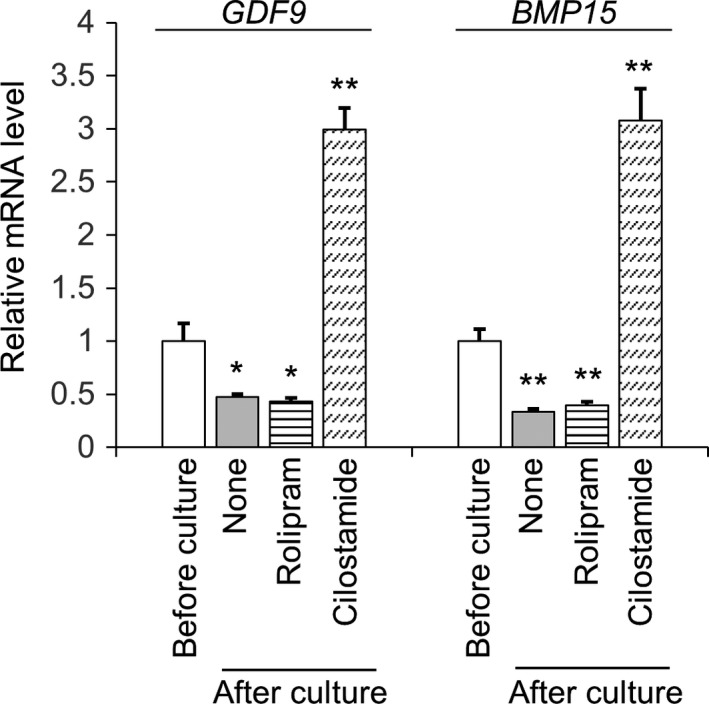
Relative expression levels of *GDF9* and *BMP15* mRNAs in bovine growing oocytes after 5 days of culture with phosphodiesterase (PDE) inhibitors were assessed by qPCR. Oocytes cultured without a PDE inhibitor were marked as “None.” Rolipram (25 µM) was used as a PDE4 inhibitor and cilostamide (5 µM) as a PDE3 inhibitor. Bovine *β‐ACTIN* was used as internal control. Data are presented as fold changes relative to the control group (before culture) and are shown as the mean ± SEM from at least three replications. *P < .05; **P < .01

In our IVG culture experiment of growing oocytes, bovine growing oocytes‐granulosa cell complexes cultured with IBMX (broad‐spectrum PDE inhibitor), cilostamide, and milrinone (PDE3 inhibitors) maintained the meiotic arrest of oocytes and gap junctional communication between oocytes and granulosa cells.^133^ In addition, these inhibitors promoted the formation of antrum‐like structures in the complexes. However, a PDE4 inhibitor, rolipram, had no effects on oocytes and granulosa cells. Since PDE3 specifically functions in oocytes, whereas PDE4 is compartmentalized in granulosa cells,^75^ it was speculated that there were some factors connecting oocyte PDE3 with granulosa cell function. Next, therefore, we examined the relative expression levels of *GDF9* and *BMP15* mRNAs in bovine oocytes by qPCR after IVG culture with these inhibitors (Figure 5). The levels of *GDF9* and *BMP15* mRNAs decreased in oocytes after culture, whereas both mRNA levels were increased by the PDE3 inhibitor. Although the mechanism connecting increased cAMP/cGMP with increased expression of *GDF9* and *BMP15* mRNAs has not been elucidated, the PDE3 inhibition probably leads to synthesis of GDF9 and BMP15 by the oocyte, with the growth factors in turn promoting the formation of antrum‐like structures by granulosa cells (Figure 2). In mouse oocyte‐ectomized complexes (cumulus cells), cGMP levels in the complexes were elevated by oocytes or GDF9.^78^ Oocyte‐derived factors and cyclic nucleotides seem to connect antiparallel bidirectional communications between oocytes and granulosa cells to make a communication loop, which ensures both oocyte growth and follicular development, including antrum formation.

## 4 CONCLUSION

In the mammalian ovary, oocyte growth and follicular development proceed in a coordinated manner in each follicle to realize the same goals of production and ovulation of fertile eggs. This process takes a long time, although oocytes maintain the meiotic arrest during their growth, while granulosa cells proliferate, differentiate, and finally form a large antrum during the follicular development. The discovery of oocyte‐derived factors (GDF9 and BMP15) has introduced a new concept of bidirectional communication between oocytes and granulosa cells, and the development of IVG culture systems has provided a robust platform to study the bidirectional communication between them. In addition to the pituitary control of follicular development, oocytes actively participate in follicular development, including proliferation and differentiation of granulosa cells *via* GDF9 and BMP15, which leads to antrum formation. On the other hand, granulosa cells also actively participate in growth and meiotic arrest of oocytes by the transfer of small molecules including cAMP and cGMP through gap junctions. Recent studies suggest that oocyte‐derived factors affect cAMP and cGMP production in granulosa cells and that these cyclic nucleotides induce GDF9 and BMP15 synthesis by oocytes. In the follicle, an oocyte and surrounding granulosa cells probably form a communication loop using these molecules, and such communication ensures the coordinated oocyte growth and follicular development.

The IVG culture for large animals including humans is still far from covering the whole process of oocyte growth. However, improvement of the IVG system in different mammalian species is essential to reveal the precise loop between oocytes and granulosa cells in the follicle. Better understanding of the communication between oocytes and granulosa cells is also essential to improve IVG systems of oocytes as an assisted reproductive technology.

## DISCLOSURES

*Conflict of interest*: The authors declare no conflict of interest.

*Human and animal rights*: This article does not contain any studies with human or animal subjects performed by any of the authors.
